# The diagnostic value and pathogenetic role of lncRNA-ATB in patients with osteoarthritis

**DOI:** 10.1186/s11658-018-0118-9

**Published:** 2018-11-27

**Authors:** Xiliang Dang, Liping Lian, Dongsheng Wu

**Affiliations:** Second Department of Orthopaedics, Wei Nan Central Hospital, Weinan, 714000 Shanxi Province People’s Republic of China

**Keywords:** Osteoarthritis, lncRNA-ATB, Proliferation, Viability, Akt signaling

## Abstract

**Background:**

In view of the roles of long non-coding RNAs (lncRNAs) in human diseases and the high incidence of osteoarthritis, we investigated the role of long non-coding RNA activated by transforming growth factor-β (lncRNA-ATB) in osteoarthritis and explored its diagnostic value for this disease.

**Methods:**

The study involved 98 patients with osteoarthritis and 76 healthy subjects. Blood was extracted from each participant and the expression of lncRNA-ATB in the serum was detected using quantitative Real Time -PCR. ROC curve analysis was performed to evaluate the diagnostic value of lncRNA-ATB for osteoarthritis. Based on the median serum level of lncRNA-ATB, patients were divided into a high-level group and a low-level group. Correlations between the serum levels of lncRNA-ATB and basic information about the patients were analyzed using the chi-square test. LncRNA-ATB overexpression in human chondrocyte cell line CHON-001 (ATCC CRL-2846) was established to study the effects on chondrocyte proliferation (using the CCK-8 assay) and viability.

**Results:**

LncRNA-ATB expression was significantly downregulated in the serum of osteoarthritis patients compared with the healthy controls, meaning this downregulation effectively distinguished osteoarthritis patients from healthy subjects. LncRNA-ATB expression in the serum was not significantly affected by the patients’ gender, age or habits, including smoking and alcohol consumption. LncRNA-ATB overexpression activated Akt signaling, promoted proliferation and increased the viability of the chondrocytes.

**Conclusion:**

We conclude that downregulation of lncRNA-ATB in serum is a reliable diagnostic marker for osteoarthritis and that this lncRNA participates in the pathogenesis of osteoarthritis by regulating the proliferation and viability of chondrocytes through the activation of Akt signaling.

**Electronic supplementary material:**

The online version of this article (10.1186/s11658-018-0118-9) contains supplementary material, which is available to authorized users.

## Introduction

Osteoarthritis is a common chronic joint disease that mainly affects middle-aged (40s to early 60s) people [1, 2]. Despite efforts made to treat and prevent this disease, its incidence is expected to further increased in the near future. Heavy physical occupational activities are major causes of this disease, especially in developing countries such as China [3, 4]. Its main pathological features are bone destruction, secondary subchondral bone hyperplasia and degenerative changes of articular cartilage. Its clinical manifestations include deformity, joint pain and dysfunction [4]. Bone fracture caused by osteoporosis is a leading cause of pain and long-term disability, or even death in some extreme cases [5].

Previous studies have shown that the onset, development and progression of osteoarthritis involve long non-coding RNAs (lncRNAs), which play pivotal roles in both physiological and pathological processes [6, 7]. For example, overexpression of the lncRNA HOTAIR in osteoarthritis cases has been found to promote disease progression [8]. By contrast, MEG3 expression is negatively correlated with VEGF in osteoarthritis patients, indicating its protective role in this disease [9].

LncRNA activated by transforming growth factor-β (lncRNA-ATB) is a newly discovered lncRNA with pivotal roles in the pathogenesis of various human malignancies [10], but its involvement in osteoarthritis remains unknown. Our study investigated the functionality of lncRNA-ATB in osteoarthritis. We found that it was downregulated in osteoarthritis patients and that it may participate in the regulation of the proliferation and viability of chondrocytes through the activation of Akt signaling.

## Materials and methods

### Subjects and specimens

The study involved 98 patients with osteoarthritis selected between January 2015 and January 2017 in Wei Nan Central Hospital. There were 54 males and 44 females, ranging in age from 32 to 69 years, with a mean age of 49 ± 8.7 years. In the same period, 76 healthy volunteers with similar age and gender distributions were selected to serve as a control group.

The healthy volunteers underwent comprehensive physical exams including laboratory tests, pulmonary function testing, urinalysis, chest x-rays, audiograms, full body CAT scanning, heart stress tests, EKGs, vascular age tests, and mammograms or prostate exams depending on gender. None of them showed any abnormities.

Whole blood (20 ml) was extracted from each participant on the day of admission to the study. Blood samples were kept at room temperature for 2 h, followed by centrifugation at 1200 g for 20 min to obtain serum. Serum samples were stored in liquid nitrogen before use.

This study was approved by the Ethics Committee of Wei Nan Central Hospital. All patients and healthy subjects signed written forms for informed consent.

### Cell line and cell culture

Human chondrocyte cell line CHON-001 (ATCC CRL-2846) was obtained from ATCC. Cells were cultured in ATCC-formulated Dulbecco’s modified Eagle’s medium (DMEM, cat. no. 30–2002) supplemented with 0.1 mg/ml G-418 and 10% heat-inactivated fetal bovine serum in an incubator (37 °C, 5% CO_2_). Cells were harvested during the logarithmic phase for subsequent experiments.

### Construction of cell line overexpressing lncRNA-ATB

LncRNA-ATB cDNA surrounded by NheI cutting sites was amplified via PCR and inserted into an NheI-linearized pEGFPC3 (Clontech) vector to establish the lncRNA-ATB expression vector. This vector has a CMV promoter.

Chondrocytes were cultured overnight to reach 70–80% confluence, and then 15 nM vectors were transfected into 4 × 10^5^ cells using Lipofectamine 2000 reagent (cat. no. 11668–019, Invitrogen). Transfection of empty pEGFPC vector was used as a negative control.

### MTT assay

Cells were harvested during the logarithmic phase to prepare a cell suspension using medium containing 10 mM tetraethylammonium (TEA). The final cell density was 4 × 10^4^ cells/ml. Then 4 × 10^3^ cells in 100 μl cell suspension were added to each well of a 96-well plate. Cells were cultured for 6 h and then 10 μl of MTT was added, followed by cell culture for an additional 4 h. A Fisherbrand accuSkan GO U*V*/Vis Microplate Spectrophotometer (Thermo Fisher Scientific) was used to measure absorbance at 570 nm.

### Cell proliferation assay

Each well of a 96-well plate was filled with 100 μl of cell suspension containing 4 × 10^3^ cells. Cells were cultured and 10 μl of CCK-8 solution (Sigma-Aldrich) was added 24, 48, 72 and 96 h later. Cells were cultured for another 4 h, and a Fisherbrand accuSkan GO U*V*/Vis Microplate Spectrophotometer was used to measure optical density at 450 nm (Fisher Scientific).

### Quantitative real-time PCR

Trizol reagent (Invitrogen) was mixed with serum or in vitro cultured cells to extract total RNA. This extraction was repeated to make sure all RNA samples had an A260-to-A280 ratio between 1.8 and 2.0 (measured using a NanoDrop 2000 Spectrophotometer, Thermo Fisher Scientific).

After synthesis of cDNA, PCR was performed with these primers:

- 5’-TCTGGCTGAGGCTGGTTGAC-3′ (forward) and 5’-ATCTCTGGGTGCTGGTGAAGG-3′ (reverse) for lncRNA-ATB;
- 5’-GACCTCTATGCCAACACAGT-3′ (forward) and 5’-AGTACTTGCGCTCAGGAGGA-3′ (reverse) for β-actin

The PCR conditions were: 95 °C for 55 s, followed by 40 cycles of 95 °C for 20 s and 60 °C for 42 s. Data were processed using the 2^-ΔΔCT^ method, and the relative expression level of lncRNA-ATB was normalized to endogenous control β-actin.

### Western blot

Total protein extraction was carried out using RIPA solution (Thermo Fisher Scientific) and the BCA assay was performed to measure protein concentration. After denaturing, protein samples (20 μg from each sample) were subjected to 12% SDS-PAGE gel electrophoresis, followed by gel transfer to PVDF membranes. Blocking was performed by incubating membranes with 5% skimmed milk for 1 h at room temperature.

After blocking, the membranes were incubated with rabbit anti- human primary antibodies of p-Akt (1:2000, ab38449, Abcam), Akt (1:2000, ab8805, Abcam) and GAPDH (1:1000, ab8245, Abcam) at 4 °C overnight. After that, membranes were washed 3 times for 5 min each time with phosphate-buffered saline (PBS). The membranes were further incubated with anti-rabbit IgG-HRP secondary antibody (1:1000, MBS435036, MyBioSource) at room temperature for 2 h. Finally, ECL (Sigma-Aldrich) was added and a MYECL Imager (Thermo Fisher Scientific) was used to detect signals. The relative expression levels of p-Akt and Akt were normalized to the endogenous control GAPDH using Image J software.

### Statistical analysis

All data were processed using SPSS19.0 (SPSS Inc.). Count data were compared using the chi-square test. Student’s t test was used for comparisons of measurement data between two groups, and analysis of variance and the LSD test were used for comparisons of measurement data between multiple groups. *p* <  0.05 indicated a difference with statistical significance.

## Results

### Expression of lncRNA-ATB in the serum of osteoarthritis patients

**Fig. 1:**
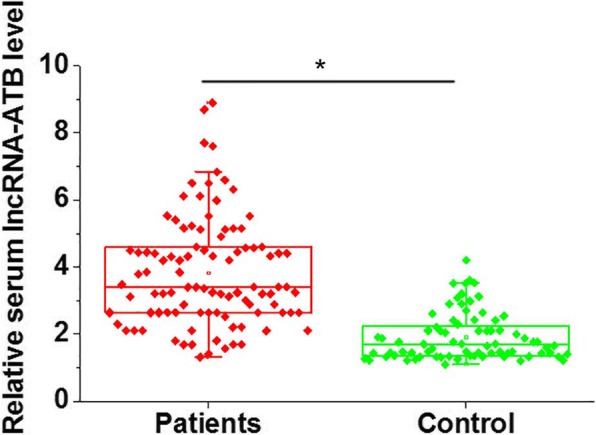
The expression of lncRNA-ATB in the serum of osteoarthritis patients and healthy controls. *p < 0.05. The levels of lncRNA-ATB in the serum of osteoarthritis patients and healthy controls were measured using quantitative Real Time-PCR. This experiment was performed in triplicate. Ct values were normalized using the 2^-ΔΔCT^ method. The results showed that serum levels of lncRNA-ATB were significantly lower in osteoarthritis patients than in healthy controls

The expressions of lncRNA-ATB in the serum of osteoarthritis patients and healthy subjects was detected using quantitative Real Time-PCR. See Additional file 1 for the data normalization using the 2^-ΔΔCT^ method. As shown in Fig. 1, the serum levels of lncRNA-ATB were significantly lower in osteoarthritis patients than in the healthy controls. These data suggest that downregulation of lncRNA-ATB is very likely to be involved in the development of osteoarthritis.

### Diagnostic values of lncRNA-ATB for osteoarthritis

**Fig. 2:**
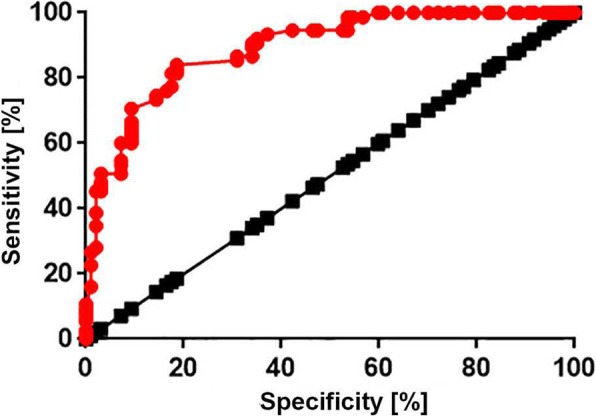
ROC curve analysis of the diagnostic value of serum lncRNA-ATB for osteoarthritis. ROC curve analysis was performed with osteoarthritis patients as true positive samples and healthy controls as true negative samples. The results showed that upregulated serum levels of lncRNA-ATB effectively distinguished osteoarthritis patients from healthy controls

ROC curve analysis was performed to evaluate the diagnostic value of lncRNA-ATB for osteoarthritis. In this analysis, the patients with osteoarthritis were used as true negative samples and the healthy controls were used as true negative samples. As shown in Fig. 2, the area under the curve (AUC) was 0.8902 (higher than 0.65), with a 95% confidence interval of 0.8430 to 0.9373 (*p* <  0.0001), indicating that serum lncRNA-ATB may serve as a diagnostic biomarker for osteoarthritis.

### Correlation between the serum levels of lncRNA-ATB and the clinical data of osteoarthritis patients

Based on the median serum level of lncRNA-ATB, the patients were divided into a high-level group and a low-level group. Correlations between the serum levels of lncRNA-ATB and clinical data were analyzed using the chi-square test. As shown in Table 1, the serum levels of lncRNA-ATB showed no significant correlations with the patients’ age, gender or habits, including smoking and drinking. However, a significant correlation was found between the serum levels of lncRNA-ATB and the progress of the disease.

**Table 1:** Correlation between the serum levels of lncRNA-ATB and clinical data for osteoarthritis patients

Items	Groups	Cases	High expression	Low expression	χ^2^	*p* value
Gender	Male	54	26	28	0.165	0.68
	Female	44	23	21		
Age	Over 45 years	52	25	27	0.164	0.69
	Under 45 years	46	24	22		
Course of disease	Over 5 years	38	29	9	17.193	< 0.01
	Under 5 years	60	20	40		
Smoking	Yes	48	27	21	1.47	0.23
	No	50	22	28		
Drinking	Yes	52	30	22	2.622	0.11
	No	46	19	27		

### LncRNA-ATB overexpression promoted the phosphorylation of Akt in cells of human chondrocyte cell line CHON-001

**Fig. 3:**
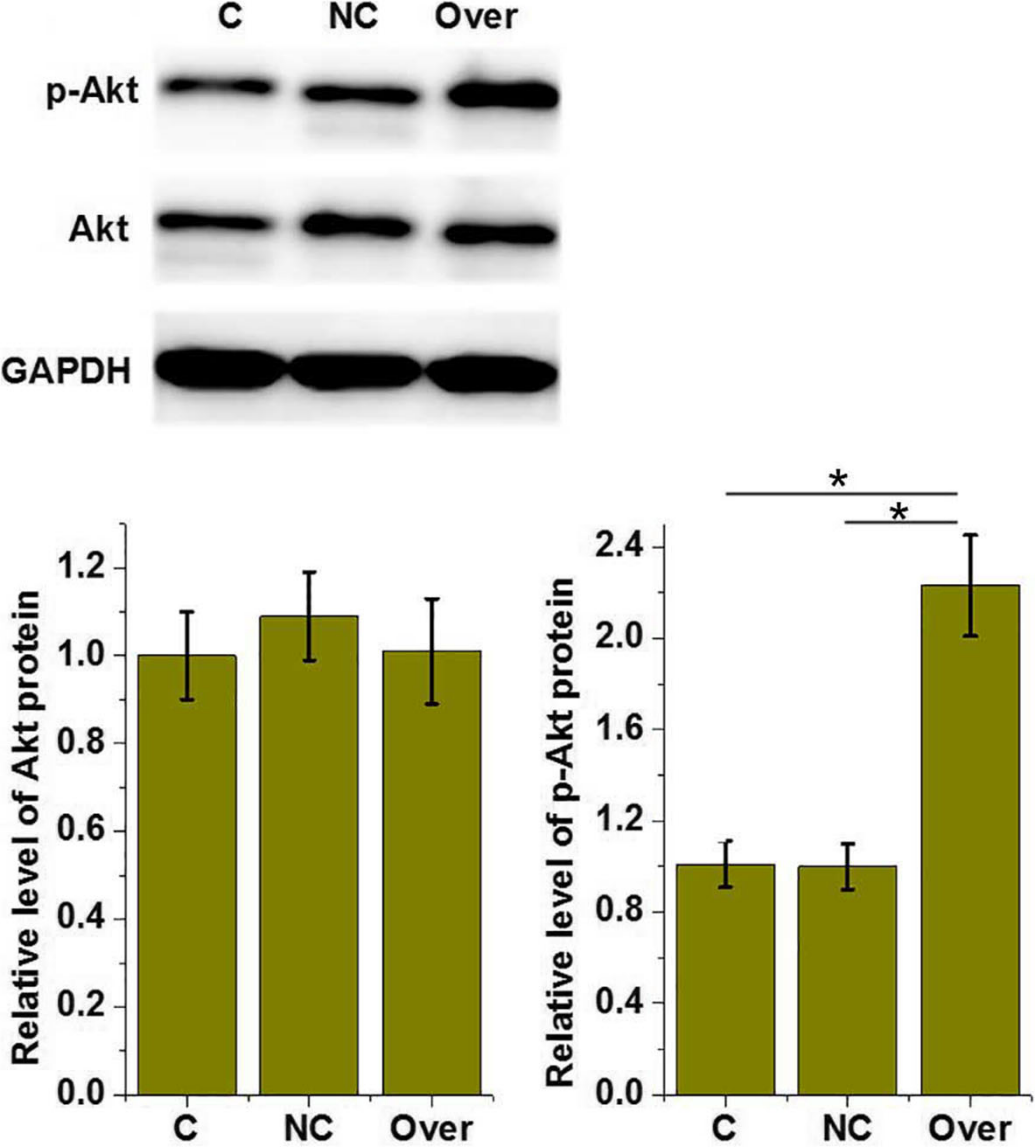
The effects of lncRNA-ATB overexpression on the expression and phosphorylation of Akt in chondrocytes. *p < 0.05. C, control; NC, negative control; Over, lncRNA-ATB overexpression. The expression and phosphorylation of Akt in chondrocytes after lncRNA-ATB overexpression was detected via western blot. LncRNA-ATB overexpression promoted the phosphorylation but not the expression of Akt

**Fig. 4:**
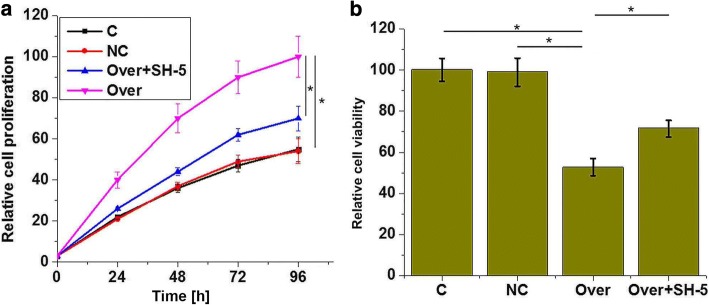
The effects of lncRNA-ATB overexpression on the proliferation and viability of chondrocytes. **a** Cell proliferation under different conditions. **b** Cell viability under different conditions. *p < 0.05. C, control; NC, negative control; Over, lncRNA-ATB overexpression. The proliferation and viability of chondrocytes after lncRNA-ATB overexpression were respectively analyzed using the CCK-8 and MTT assays. LncRNA-ATB overexpression promoted the proliferation and increased the viability of chondrocytes

Akt signaling has been proven to be involved in the pathogenesis of osteoarthritis [11]. In our study, lncRNA-ATB overexpression showed no significant effect on the expression level of Akt in the cells of human chondrocyte cell line CHON-001 (Fig. 3). However, compared with the control cells and negative control cells (empty vector transfection), the phosphorylation level of Akt was significantly higher in cells with lncRNA-ATB overexpression (*p* < 0.05, Fig. 4). These data suggest that lncRNA-ATB overexpression can activate Akt signaling in chondrocytes.

### Effects of lncRNA-ATB overexpression on the proliferation and viability of chondrocytes

As shown in Fig. 4a, lncRNA-ATB overexpression significantly promoted the proliferation of chondrocytes (*p* < 0.05), while treatment with the Akt signaling inhibitor SH-5 (10 nM, Santa Cruz Biotechnology) significantly reduced the enhancing effects of lncRNA-ATB overexpression on the proliferation of chondrocytes (p < 0.05).

In addition, lncRNA-ATB overexpression significantly increased the viability of chondrocytes, while treatment with the Akt signaling inhibitor SH-5 significantly reduced the enhancing effects of lncRNA-ATB overexpression on the viability of chondrocytes (Fig. 4b, *p* < 0.05).

These data suggest that lncRNA-ATB overexpression can promote the proliferation and increase the viability of chondrocytes by activating Akt signaling.

## Discussion

Studies in last several years have shown that the development of osteoarthritis is accompanied by abnormal expression of certain genes. This includes changes in the expression of various long non-coding RNAs (lncRNAs). Zhang et al. showed that the lncRNA HOTAIR was significantly upregulated in patients with osteoarthritis compared with healthy subjects, and that this increased expression closely correlated with the progression of the disease [8]. By contrast, MEG3 expression is downregulated in osteoarthritis patients and negatively correlates with the expression level of VEGF [9].

The expression pattern of lncRNA-ATB has been examined in several types of human malignancy. Significantly upregulated expression of lncRNA-ATB in tumor tissues not only promotes tumor growth and progression, but also induces the development of cancer cell resistance to anti-tumor drugs [12, 13]. However, the expression pattern of lncRNA-ATB in patients with osteoarthritis is unknown.

In our study, significantly lower serum levels of lncRNA-ATB were found in patients with osteoarthritis than in healthy controls. Those data suggest that downregulation of lncRNA-ATB is very likely involved in the pathogenesis of osteoarthritis**.**

The development of human diseases is usually accompanied by changes in certain substances in the blood, and the detection of those substances may provide references for the diagnosis of certain diseases [14]. In this study, ROC curve analysis showed that serum lncRNA-ATB can be used to effectively distinguish patients with osteoarthritis from healthy controls. Furthermore, a significant correlation was found between the serum level of lncRNA-ATB and the progress of the disease.

It is known that alcohol abuse [15] and tobacco consumption [16] can affect the expression of certain genes and lncRNAs, which may affect the accuracy of lncRNA biomarkers in the diagnosis of diseases. In addition, aging and gender also have significant effects on the transcription profile of human genome [17, 18].

In our study, chi-square analysis showed that serum levels of the lncRNA-ATB had no significant correlations with age, gender or habits, including smoking and drinking.

These data suggest that serum lncRNA-ATB is an accurate and effective biomarker for osteoarthritis. It is worth mentioning that abnormal expression of lncRNA-ATB has been observed in multiple human diseases, which may affect the specificity of serum lncRNA-ATB in the diagnosis of osteoarthritis. Therefore, combined used of multiple biomarkers is needed to improve diagnostic specificity.

Akt signaling plays a pivotal role in the development of osteoarthritis, and the activation of Akt signaling has been proven to be responsible for the occurrence of inflammatory responses in chondrocytes derived from the animal model of osteoarthritis [19, 20]. In this study, lncRNA-ATB overexpression significantly promoted the phosphorylation of Akt in human chondrocytes. In addition, lncRNA-ATB overexpression significantly promoted the proliferation and increased the viability of chondrocytes, while Akt inhibitor treatment reduced the effects of lncRNA-ATB overexpression on the proliferation and viability of chondrocytes. These data suggest that lncRNA-ATB overexpression can promote the proliferation and increase the viability of chondrocytes through the activation of the Akt signaling pathway.

## Conclusion

Based on our results, which are admittedly for a relatively small sample size, serum lncRNA-ATB may serve as a promising biomarker for osteoarthritis. Future studies with a larger sample size are needed to further confirm our conclusions.

## Additional file

Additional file 1: Data normalization using 2-ΔΔCT method. (XLS 62 kb)

## Abbreviations

AUC: Area under the curve
BCA: Bicinchoninic acid assay
CAT: Computerized axial tomography
CCK-8: Cell Counting Kit-8
ECL: Electrochemiluminescence
EKGs: Electrocardiograms
GAPDH: Glyceraldehyde 3-phosphate dehydrogenase
LncRNA: Long non-coding RNA
LncRNA-ATB: LncRNA activated by transforming growth factor-β
LSD: Least significant difference
MTT: (3-(4,5-Dimethylthiazol-2-yl)-2,5-diphenyltetrazolium bromide
PBS: Phosphate-buffered saline
PVDF: Polyvinylidene difluoride
RIPA: Radioimmunoprecipitation assay buffer
ROC: Receiver operating characteristic
SDS-PAGE: Sodium dodecyl sulfate–polyacrylamide gel electrophoresis
